# The Effects of Baicalin and Baicalein on Cerebral Ischemia: A Review

**DOI:** 10.14336/AD.2017.0829

**Published:** 2017-12-01

**Authors:** Wei Liang, Xiaobo Huang, Wenqiang Chen

**Affiliations:** Department of Traditional Chinese Medicine, Xuanwu Hospital, Capital Medical University, Beijing, 100053, China; Department of Traditional Chinese Medicine, Xuanwu Hospital, Capital Medical University, Beijing, 100053, China; Department of Traditional Chinese Medicine, Xuanwu Hospital, Capital Medical University, Beijing, 100053, China^#^These authors equally contributed to this work.

**Keywords:** baicalin, baicalein, cerebral ischemia, neuronal protection

## Abstract

Ischemic stroke, producing a high mortality and morbidity rate, is a common clinical disease. Enhancing the prevention and control of ischemic stroke is particularly important. Baicalin and its aglycon baicalein are flavonoids extracted from *Scutellaria baicalensis*, an important traditional Chinese herb. In recent years, a growing body of evidences has shown that baicalin and baicalein could be effective in the treatment of cerebral ischemia. Pharmacokinetic studies have shown that baicalin could penetrate the blood-brain barrier and distribute in cerebral nuclei. Through a variety of *in vitro* and *in vivo* models of ischemic neuronal injury, numerous studies have demonstrated that baicalin and baicalein have salutary effect for neuroprotection. Especially, the studies on the pharmacological mechanism showed that baicalin and baicalein have several pharmacological activities, which include antioxidant, anti-apoptotic, anti-inflammatory and anti-excitotoxicity effects, protection of the mitochondria, promoting neuronal protective factors expression and adult neurogenesis effects and many more. This review focuses on the neuroprotective effects of baicalin and baicalein in ischemia or stroke-induced neuronal cell death. We aimed at collecting all important information regarding the neuroprotective effect and its pharmacological mechanism of baicalin and baicalein in various *in vivo* and *in vitro* experimental models of ischemic neuronal injury.

The genus *Scutellaria (Lamiaceae)*, commonly known as ‘Skullcaps’, includes about 350 species [1] which are widespread in temperate regions and tropical mountains, including Europe, North America and East Asia [2]. According to Flora of China, there are almost 102 varieties of scullcap in China. Chinese Pharmacopoeia (2005) contained only one species, *Scutellaria baicalensis Georgi* (Huang-qin or Chinese skullcap), as a genuine Chinese medical material in Hebei Province of China. This plant is a perennial herb with fleshy roots, branched stems, papery leaves, purple-red to blue flowers, and black-brown ovoid nutlets. The root of this plant (*Radix scutellariae*) is an important traditional Chinese medicine which was first recorded in Shennong’s Classic of Materia Medica (Shen Nong Ben Cao Jing) in ca. 100 BC and has been widely used in China, Korea and Japan for thousands of years [3]. In addition to the genuine Chinese medical materials, some species of Scutellaria also have been used as Huang-qin in different regions. For instance, *Scutellaria Viscidula Bge*, *Scutellaria rehderiana Diels, Scutellaria Amoena C.H. Wrigh, Scutellaria Likiangensis Diels* and *Scutellaria hyperifolia Levi*.

The chemical compounds of the genus Scutellaria have been studied since 1889. Since then, more than 295 compounds have been isolated from 35 species, in which phenolic compounds (flavonoids, phenylethanoid glycosides) and terpene compounds (iridoid glycosides, diterpenes and triterpenoids) are the two main groups of constituents, and the plants also contain alkaloids, phytosterols and polysaccharides among others [4].

Baicalein (5,6,7-trihydroxyflavone) and baicalin (syn. baicalein7-O-β-D-glucuronic acid) (Fig. 1) [5] are the principal components found among 30 other flavonoid derivatives in the roots of *Scutellaria baicalensis Georgi*. Baicalin and its aglycon baicalein have been attracting growing interest from pharmaceutical industries because of their excellent biological action. Although there are structural similarities between these two flavonoids and they can convert to each other during the metabolism in the body, they may exert different effects on mammalian cells [6]. Numerous studies have demonstrated that they have anti-viral [7], anti-oxidative [8], anti-tumor [9], anti-thrombotic [10], anti-apoptotic properties, and neuronal protection effects on cerebral ischemia/reperfusion injury [11].

**Figure 1.**
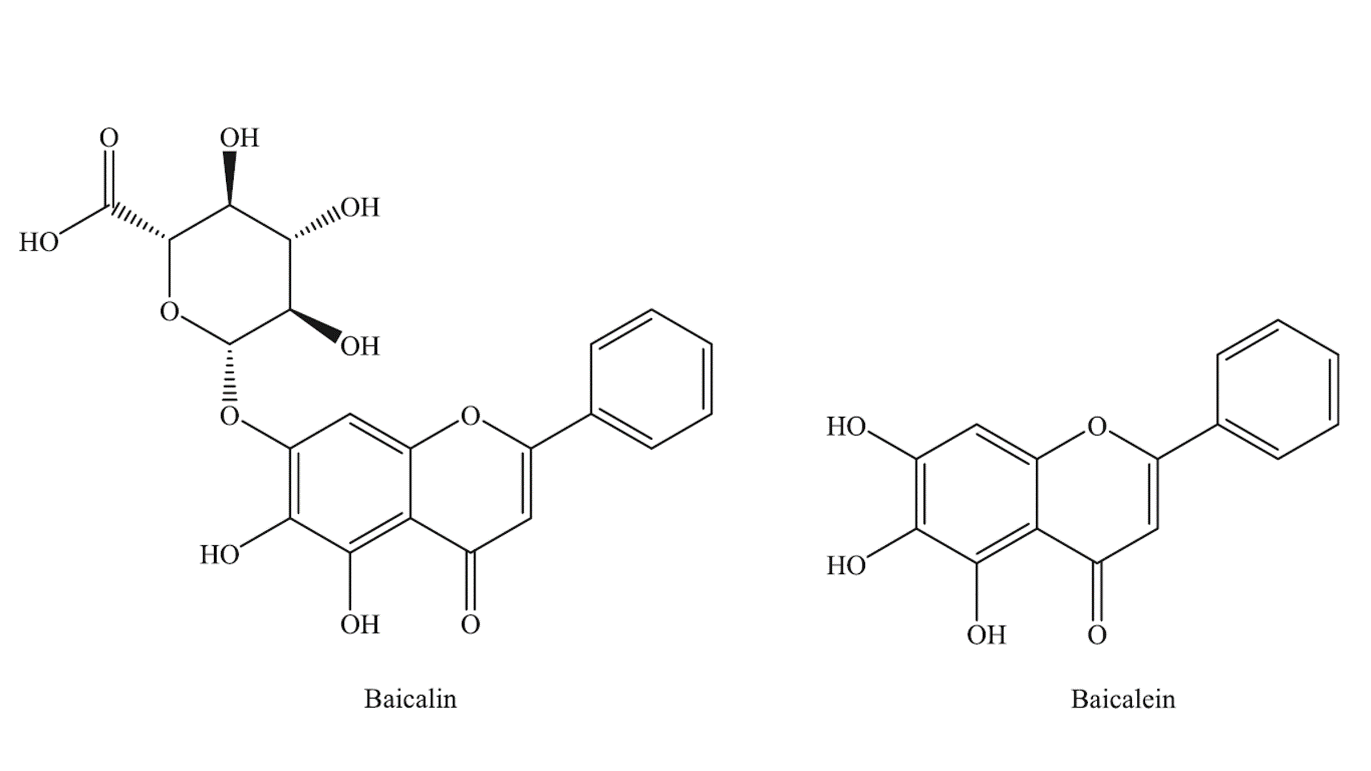
Structures of baicalein and baicalin.

Ischemic stroke is the third most common cause of death and the leading cause of disability worldwide in adults [12]. The World Health Organization estimates that 5-6 million people die from stroke worldwide each year. Ischemic stroke has two major potential therapeutic strategies: thrombolytic therapy and neuroprotective therapy. The currently available treatment for acute ischemic stroke is the administration of the thrombolytic agent, tissue-plasminogen activator. However, only 1-2% of patients have the chance to receive thrombolytic therapy mainly due to the short therapeutic time window [13]. Therefore, it is an essential task to explore new therapeutic approaches in this area. Accumulating evidence has demonstrated that cerebral ischemia/reperfusion injury are related to excitotoxicity, excessive formation of reactive oxygen species (ROS), mitochondrial dysfunction, inflammatory response, neuronal apoptosis [14], and Blood-brain barrier permeability and so on [15]. In this review, we focus on the discussion of the protective effects of baicalin and baicalein on cerebral ischemia.

## Biopharmaceutics and pharmacokinetics

Based on the calculations from ALOGPS (version 2.1), the log P values of baicalein and baicalin are 2.59 and 0.68. Solubility of Baicalein and baicalin obtained from the same software calculation are 0.15 g/l and 1.72 g/l, respectively. Baicalein exhibit a higher log P value and lower solubility than its glucuronides, which indicates that baicalein may have higher intestinal permeability than baicalin [16]. The biopharmaceutical profiles of baicalein and baicalin have been investigated using *in vitro* and *in situ* models such as rat intestinal perfusion and Caco-2 cell monolayer models. It was found that baicalein, rather than baicalin could pass through the intestinal epithelium efficiently [17]. Baicalein is able to permeate easily through the monolayer from the apical (lumen) to the basolateral (blood) side due to its high lipophilicity and low molecular weight and lack of transporters. However, baicalin exhibited limited permeability possibly due to its relatively high hydrophilicity and larger molecular weight. Using *in situ* jejunal loop technique and *in vitro* jejunal everted sac experiments, it was found that baicalein was extensively metabolized into baicalin in intestinal mucosal cells and baicalin was excreted into intestinal lumen by multidrug resistance associate protein 2 (MRP2) [18].

### Absorption

In situ perfusion experiments were performed in rats with and without the ligation of the bile duct. The results showed that baicalin was moderately absorbed in the stomach, but poorly absorbed from the small intestine and colonic regions. However, baicalein was well absorbed from the stomach and small intestine, but absorption was somewhat limited from the colon. The use of bile duct ligation helped to clarify the role of biliary excretion of baicalin and the importance of the two agents to keep a balance of the systemic levels of both baicalin and baicalein. It was concluded that baicalein was the more preferred species for oral absorption due to the body dynamics of a more complete absorption of baicalein and restoration of baicalin in the systemic circulation by conjugative reactions of baicalein. The circulating baicalin would be expected to re-enter the gastrointestinal tract via the biliary excretion mechanism [19]. Another study also showed that baicalin itself cannot be absorbed directly across the intestine and was firstly hydrolyzed into baicalein by intestinal bacteria [20]. The enzymes in gastrointestinal (GI) tract such as beta-glycosidase or lactase phlorizin hydrolase (LPH) also can hydrolyze baicalin [21].

### Distribution

Enterohepatic recirculation of the baicalin conjugates may be an important distribution phenomenon for baicalin, which has been confirmed by the multi-peak phenomenon of the plasma concentration-time curve after both oral and intravenous routes of dosing in rats [22]. The specific distributions of baicalin to tissues/organs have not been reported, it seems that baicalin is distributed to many tissues/organs in the body. The *in vitro* protein binding of baicalin has been studied in human plasma and was found to range from 86% to 92%, suggesting that it is no problem for baicalin releasing from plasma protein binding [23]. An in vivo microdialysis sampling method coupled with ultra-performance liquid chromatography-tandem mass spectrometry (UPLC-MS/MS) was employed for continuous simultaneous monitoring of unbound baicalin in rat blood and brain. Microdialysis probes were inserted into the jugular vein and brain cerebrospinal fluid of Sprague-Dawley rats, following administration of baicalin at doses of 24mg/kg via the caudal vein, samples were collected every 20min and injected directly into the UPLC-MS/MS system. The time-concentration curves of baicalin in rat blood and brain were obtained. It was concluded that baicalin can cross the BBB and distribute into the CSF quickly and reach its peak concentration of 344 g/l about 30 min after the i.v. administration of 24 mg/kg [24]. Zhang et al. investigated the pharmacokinetic process of baicalin in normal rat blood and cerebral nuclei including cortex, hippocampus, striatum, thalamus and brain stem with a newly established reverse-phase HPLC method after intravenous administration of baicalin-enriched Scutellariae Radix extract. The results indicated that the distribution of baicalin into brain was a subsequent process and baicalin tends to accumulate in the striatum, thalamus and hippocampus with the exhibition of large area under the concentration-time curve and mean residence time values [25]. These evidences support the therapeutic effects of baicalin on the central nervous system.

### Metabolism

The major route of metabolism for baicalein in plasma and urine is conjugated metabolism. A study showed that the intact levels of baicalein in plasma were negligible following oral dosing of baicalein; however, the conjugates of baicalein with glucuronides and sulfate appeared in the plasma. Following intravenous dosing of baicalein, about 76% of the baicalein was converted into the conjugated forms. On the other hand, when baicalin was orally administered, the intact baicalin and glucuronide and sulfate conjugates of baicalein were observed in the serum [26]. It was observed that baicalin was subjected to extensive metabolism, via conjugative reactions, in both ileum and jejunum regions of the rats. A higher loading dose of baicalin could cause saturation in the metabolism that is an evidence of baicalin undergoing first-pass metabolism [27].

### Excretion

The excretion of baicalin were investigated in a multi-drug resistance associated protein 2-deficient (Mrp2-deficient) and normal rats. The peak concentration and area under the curve (AUC) value for baicalin in Mrp2-deficient rats were five-fold and eight-fold higher than the relative values in normal rats after oral administration of baicalein. The biliary excretion and systemic exposure for baicalin in Mrp2-deficient rats showed a four-fold reduction and 30-fold elevation compared with normal rats after administration of baicalein. This work confirmed the biliary excretory pathway and sinusoidal efflux mechanism for baicalin [28]. Liu et al. also confirmed that the glucuronide and sulfate conjugates of baicalin were excreted into small intestine from biliary pathway [29]. Baicalein glucuronide and sulfate conjugates also could be excreted from renal, for example, a study showed that up to 7.2% of the administered dose of Scutellariae radix in humans appeared in urine. Therefore, the renal route may be a minor pathway for the excretion of conjugates of baicalein [26].

Some studied showed baicalein enters the bloodstream in the form of glucuronide or sulfate conjugates and have very low oral bioavailabilities [30]. The permeability coefficient of baicalein was high in the Caco-2 cell model and in the rat in situ intestinal perfusion model, suggesting a good intestinal permeability. But baicalein also underwent fast and extensive metabolism after either its p.o. or i.v. administration. As a result, the bioavailability of baicalein was quite low [22].

A study investigated the pharmacokinetic effects of baicalin on cerebral ischemia-reperfusion (I/R) after its administration in rats. In this experiment, the cerebral I/R rats were induced by occluding the bilateral carotid arteries of normal rats for 2 h, followed by reperfusion. The model animals were immediately administrated with baicalin (90 mg/kg). As control, the same dose of baicalin was injected to the normal rats. Plasma samples were collected at different times to construct pharmacokinetic profiles. In normal rats, the major parameters of distribution half-life, elimination half-life, area under the plasma concentration-time (AUC), apparent volume of distribution (Vd), and clearance (CL), estimated by an open two-compartmental model, were 0.8868 min, 26.0968 min, 149.6204 mg/min·L, 4.765 L/kg, and 0.5776 L/ kg·min, respectively. However, in I/R rats, the corresponding parameters were 2.084 min, 34.4998 min, 260.0188 μg·min/L, 5.9376 L/kg, and 0.334 L/(kg·min), respectively. The cerebral I/R could significantly increase AUC and Vd values, decrease CL values, and prolong the terminal half-life of baicalin [31].

A sensitive and specific HPLC method was developed to analyze baicalin in rat plasma. Zeng et al. compared the pharmacokinetics of baicalin after oral administration of pure baicalin and Huang-Lian-Jie-Du-Tang (HLJDT) decoction, which has been used in the therapy of cerebrovascular disease in China and other Asian countries in the clinical practice. All the rats were divided into two groups, MCAO and sham-operated rats. Each group contained two subgroups: HLJDT decoction (10.00 g/kg decoction extract, containing baicalin 400.00 mg/kg) and pure baicalin subgroup (400.00 mg/kg) receiving the gavages at a dosage according to body weight. The results indicated that the pharmacokinetics of baicalin in rat plasma was non-linear and no matter in MCAO or sham-operated rats, pure baicalin had shown better absorption than HLJDT decoction. The MCAO rats showed better, quicker absorption of baicalin than sham-operated rats for both of pure baicalin and HLJDT decoction. This study concluded that in the pathologic condition, baicalin had a better absorption effect, and it proves the rationality of using it in cerebrovascular disease, which would improve the therapeutic efficacy [32].

The transferrin receptors were strongly expressed on the apical side of the blood-brain barrier and the solid lipid nanoparticles modified by OX26 (anti-transferrin receptor antibody) could enhance drug penetration across the BBB. The pharmacokinetic study on baicalin-loaded PEGylated cationic solid lipid nanoparticles modified by OX26 antibody (OX26-PEG-CSLN) was carried out in a MCAO rat model. The results showed the AUC value of OX26-PEG-CSLN was 5.69-fold higher than that of the baicalin solution (P < 0.05) and the Cmax value of OX26-PEG-CSLN was 6.84-fold higher than that of the baicalin solution (P < 0.05). Therefore, OX26-PEG-CSLN could improve uptake of baicalin across the BBB into the brain, and elevated bioavailability of baicalin in cerebral spinal fluid of rats under the cerebral ischemia-reperfusion injury [33].

## Drug-drug interaction

A clinical investigation to evaluate the induction of cytochrome P450 (CYP) 2B6 caused by baicalin has been carried out. All subjects were also genotyped for the various alleles of CYP2B6. The results showed that daily administration of baicalin could significantly induce the expression of cytochrome P450 (CYP) 2B6 and the induction of CYP2B6 altered the bioavailability at a pre-systemic level. Thus, the long-term co-administration of baicalin with other CYP2B6 substrates need to be closely monitored [34]. Jang et al. found that baicalin could inhibit CYP2E1 expression in liver. This finding suggests that baicalin may influence the metabolism of several drugs which are dependent on CYP2E1 isozyme, such as chlorzoxazone [35]. The effects of antibiotics on the absorption of baicalin and baicalein were investigated in rats. The results found that following treatment with aminoglycoside, the absolute bioavailability of baicalin was reduced by nearly 40-45% as compared with the untreated rats. However, aminoglycoside pre-treatment did not alter the absorption of baicalein [36]. Other studies also showed that baicalin and/or baicalein could interacted with transporter-related drugs, such as rosuvastatin, SN-38 (7-ethyl-10-hydroxycamptothecin) and other drugs including cyclosporine A, quinidine, and SKF-525A [37].

### Ischemia/reperfusion injury cell models

A study showed that baicalein could significantly promote mouse hippocampal HT22 cell survival with an estimated dose of 2 μM for 50% cell survival following incubation in the presence of iodoacetic acid (20 μM), an irreversible inhibitor of the glycolytic pathway that causes the free radical production, lipid peroxidation and cell death [38]. In another *in vitro* study, oxygen and glucose deprivation (OGD) was used to mimic ischemic insult in primary cultured cortical neurons. In this study, baicalein was reported for the first time to protect cortical neuronal cells from OGD [39]. The protective effect of baicalin and its three analogs on neuronal cell PC12 following oxygen-glucose deprivation (OGD) were investigated, and it was found that baicalin and its three analogs did protect neurons from OGD damage [40]. In hippocampal neurons and SH-SY5Y cells OGD models, baicalin also showed neuronal protective effects [41].

## Ischemia/reperfusion injury in animal models

Using a rabbit small clot embolic stroke model (RSCEM), the effects of baicalein on animal model’s behavioral deficits associated with multiple infarct ischemic events were studied. Baicalein (100 mg/kg, s.c.) injected 5 min and 60 min post-embolization significantly (P<0.05) improved behavioral function [38]. The effects of baicalein on cognitive and motor ability impaired by chronic cerebral hypo-perfusion in rats were investigated. Rats subjected to permanent bilateral common carotid artery occlusion experienced cognitive deficits. Results showed that baicalein could alleviate cognitive and motor impairments in this animal model [42].

Cui et al. investigated the potential neuroprotective effects of baicalein on male, Sprague-Dawley rats subjected to permanent middle cerebral artery occlusion (MCAO). Baicalein was administered intravenously immediately after cerebral ischemia. At 24 h after MCAO neurological deficit, brain water content and infarct sizes were measured. The results showed that baicalein improved neurological deficit, reduced brain water content and infarct sizes [43]. In another study, either permanent or transient (2 h) middle cerebral artery occlusion (MCAO) was induced in rats. The results showed that permanent MCAO led to larger infarct volumes in contrast to transient MCAO and only in transient MCAO, baicalein administration significantly reduced infarct size [39]. Tu et al. and Xue et al. investigated the effects of baicalin on the same MCAO rat model. Twenty-four hours after reperfusion, the neurological deficit was scored and the cerebral infarct area and infarct volume were measured. Hematoxylin and eosin (HE) staining was performed to analyze the histopathological changes of cortex and hippocampus neurons. These studies found that baicalin reduced the neurological deficit scores, cerebral infarct area and infarct volume [44, 45].

Cao et al. evaluated the neuroprotective effects of baicalin in gerbils subjected to transient global cerebral ischemic-reperfusion injury. In this study, baicalin at doses of 50, 100 and 200 mg/kg was intraperitoneally injected into the gerbils immediately after cerebral ischemia. Seven days after reperfusion, H&E staining was performed to analyze the CA1 pyramidal damage in hippocampus. Histopathological examination showed that the administration of baicalin (100 and 200 mg/kg) significantly attenuated ischemia-induced neuronal cell damage [46].

Cheng et al. invested the effects of baicalin on the spatial learning ability of global ischemia/reperfusion rats. Sprague Dawley rats were divided into three groups including sham group; global cerebral ischemia/reperfusion group; and global cerebral ischemia/reperfusion baicalin treatment group. A Morris water maze test was used to assess learning and memory, H&E staining was conducted for pathomorphology. The results showed that baicalin improved the learning and memory of global cerebral ischemia/reperfusion rats [47]. Wang et al. also invested the effects of baicalin on learning and memory impairment after global cerebral ischemia/reperfusion in gerbil. In this study, the Morris water maze test showed that baicalin significantly improved learning and memory impairment [41].

It is very important to have a certain time window for proposal medicine in stroke patients. Cheng et al. investigated the effects of baicalin on an ischemia-reperfusion-induced brain injury model in rats via middle cerebral artery occlusion (MCAO). Baicalin was injected at different time points (0, 2, 4, and 6 h) after the MCAO was applied. The results found that baicalin can significantly decrease brain infarction and improve neurological function within a time window of 4 h, which indicates a promising clinical use [48].

Vascular dementia (VD), which is characterized by progressive intellectual decline produced by ischemia hypoxia or hemorrhage brain lesion. Brain hypo-perfusion is believed to be a critical factor on the occurrence of VD. Permanent occlusion of the bilateral common carotid arteries (2VO) induced a state of chronic and moderate ischemia associated with cognitive alterations and neuronal degeneration in rats, and this animal model allowed scientists to understand pathophysiology of chronic cerebrovascular disorders. It was found that baicalein (2 or 4 mg/kg/day, i.p.) significantly improved 2VO-induced cognitive deficits and neuropathological changes. Biochemical and histological examinations revealed that baicalein reduced the increased activities of superoxide dismutase (SOD) and malondialdehyde (MDA), and attenuated the decreased activities of glutathione peroxidase (GPx) and catalase in 2VO rats [49].

## Oxidative stress

Under normal physiological condition, appropriate amounts of reactive oxygen species (ROS) play a crucial role during some important physiological processes, and it can be quickly cleaned by some antioxidant enzymes such as superoxide dismutase (SOD), glutathione peroxidase (GSH-PX) and antioxidants, such as glutathione (GSH). On the other hand, excess of ROS generated during global ischemia has been regarded as an important factor leading to the delayed neuronal cell death. Some radical scavengers and antioxidants have been shown to be effective in ischemic stroke therapy [50]. Cao et al. examined the effects of baicalin on anti-oxidative enzymes, such as SOD, GSH-PX, non-enzymatic scavenger glutathione and the content of malondialdehyde (MDA) in hippocampus for a transient global cerebral ischemic-reperfusion injury in gerbils and found that MDA level was significantly reduced and the activities of SOD and GSH as well as GSH-PX were obviously elevated in baicalin-treated groups. This finding indicated that the neuroprotection action of baicalin against ischemia/reperfusion injury was related to its antioxidant property [46].

Cheng et al. also investigated the effects of baicalin on ROS generation, MDA content, SOD activity, and NADPH oxidase activity in MCAO mouse brain tissue. Their results showed that baicalin significantly reduced ROS production and decreased MDA concentration in the mouse model brains. Furthermore, this group also investigated the effect of baicalin on a H_2_O_2_-induced primary neuronal injury *in vitro* model and found that treatment with baicalin significantly increased the cell viability, reduced the LDH leakage, and enhanced the SOD activity. The direct effects of baicalin on the scavenging activities of hydroxyl radicals, superoxide anions, and DPPH radicals and the inhibition of xanthine oxidase also have been investigated and the results showed that baicalin demonstrated beneficial effects on both direct free radical scavenging activities and the inhibition of xanthine oxidase [48].

12/15-lipoxygenase (12/15-LOX) is related to brain tissue damage subjected to oxidative stress. Neuronal 12-LOX leads to the influx of Ca^2+^, the production of peroxides, and ultimately to cell death. A study found that baicalein could effectively reduce 12/15-LOX expression in rat MCAO model [43].

Mitochondria dysfunction induced by chronic cerebral hypo-perfusion played a key role in the generation of ROS, resulting in oxidative damage. Baicalein could decrease mitochondria ROS production, in accordance with its improvements on membrane potential level, oxidative phosphorylation process, mitochondrial swelling degree, B-celllymphoma-2/Bcl-2-associated X protein (Bcl-2/Bax) ratio and cytochrome c release. These data indicated that baicalein might have therapeutic potential for the treatment of dementia caused by chronic cerebral hypoperfusion [42].

Phosphatidylinositol 3-kinase/Protein Kinase B (PI3K/Akt) regulates the survival response against oxidative stress-associated neuronal apoptosis [51]. In the central nerve system, increased Akt activity contributes to the neuroprotection induced by hypoxic preconditioning [52]. Phosphorylation of Akt (Ser473) is required for Akt activation. Activated Akt promotes cell survival and suppresses apoptosis by phosphorylation or inhibition of several downstream substrates, including glycogen synthase kinase-3 beta (GSK3β) [53]. Bcl-2-associated death promoter (BAD) is also the target of Akt. Under cell stress, the loss of Akt activity leads to BAD dephosphorylation and translocation to mitochondria, where it binds with Bcl-2 and activates the mitochondrial cell death pathway to release cytochrome c into cytosol. Liu et al. found that baicalein treatment could enhance the reduced Akt phosphorylation after oxygen and glucose deprivation/reperfusion (OGD/R) in cortical neuron. Pharmacologic inhibition PI3K or silencing Akt expression impaired the ability of baicalein to protect against OGD/R-induced cortical neurons death. At the same time baicalein increased phosphorylation of GSK3beta and BAD, and inhibited OGD/R-induced loss of Bcl-2 from mitochondria. Cytochrome c release in cytosol was sequentially blocked. The biological effects of Akt are dependent on the balance between the activity of PI3K and Phosphatase and tensin homologue (PTEN). PTEN is a major negative regulator in the PI3K/Akt signaling pathway. Phosphorylation of PTEN results in its inactivation, and then protected brain tissue from ischemic damage [54]. In OGD/R-treated cortical neurons, PTEN is rapidly dephosphorylated after OGD and this dephosphorylation is reversed by baicalein. Therefore, it seems that baicalein could cause PTEN to lose its activity and, increase AKT activity and then inhibit the downstream mediated apoptotic cell death in ischemia/reperfusion [39].

Reactive nitrogen species (RNS) is critical neurotoxic factors in cerebral ischemia-reperfusion injury. It can trigger numerous molecular cascades, resulting in neuronal death. Peroxynitrite is a representative RNS, which is produced from the reaction of nitricoxide and super oxide anions, but has much higher activity than its parent radicals. Peroxynitrite can easily cross plasma membrane and oxidize many intracellular molecules including lipid, DNA and proteins [55]. Peroxynitrite decomposition catalyst (PDCs) could reduce nitricoxide, super oxide anions and peroxynitrite and decreases its decomposition to other reactive intermediates. PDCs revealed to attenuate infarct volume, neuronal death and BBB permeability in focal cerebral ischemic rats [56]. Xu et al. explored the neuroprotective mechanisms of baicalin against RNS related neurotoxic factors in a chemical system and cell model. In the chemical systems, electron paramagnetic resonance (EPR) spin trapping experiments and mass spectrometry (MS) studies were conducted to evaluate the scavenging effects of baicalin on superoxide and nitricoxide and the reaction of baicalin and peroxynitrite. Baicalin revealed a strong antioxidant ability by directly scavenging superoxide and reacting with peroxynitrite. In cellular experiments, the effects of baicalin against extraneous and endogenous peroxynitrite mediated neurotoxicity in SH-SY5Y cells treated with peroxynitrite donor, synthesized peroxynitrite and exposed to oxygen glucose deprivation and reoxygenation (OGD/RO) were investigated. The results showed that baicalin could protect the neuronal cells from extraneous and endogenous peroxynitrite-induced neurotoxicity [57].

## Apoptosis

Apoptosis can be triggered by numerous mediators including receptor-mediated signals, withdrawal of growth factors, and environmental agents. Caspases are a family of cystein-dependent proteases in the initiation and execution of cell apoptosis. It has been reported that oxidative stress induces the release of mitochondrial cytochrome c and other apoptogenic proteins from the mitochondrial inter-membrane space into the cytosol. These proteins then activate pro-caspase-3, which leads to mitochondrial dependent apoptosis [58]. The B-cell lymphoma-2 (Bcl-2) family, consisting of anti-apoptotic (e.g. Bcl-2) and proapoptotic members, plays an important role in the regulation of cell death. Survivin, an inhibitor of apoptosis, is suggested to be crucial in controlling the initiation of the upstream anti-apoptotic mechanism that leads to the mitochondrial-dependent apoptosis. Signaling through the Janus kinase/Signal transducer and activator of transcription (JAK/STAT) pathway is initiated when a cytokine binds to its corresponding receptor. Bcl-2 and caspase-3 are important STAT3 target genes. The previous study indicates that ischemia acts synergistically to promote activation of STAT3 and STAT3-dependent transcription of survivin in insulted CA1 neurons and identifies STAT3 and survivin as potentially important therapeutic targets in an in vivo model of global ischemia [59]. A study showed that baicalin could up-regulate the expression of p-STAT3, survivin and Bcl-2 obviously in PC12 cells exposed to H_2_O_2_ for 12 hours, while caspase-3 expression was down-regulated. The results suggest that the effects of baicalin on anti-apoptosis are related to the activation of the JAK/STAT3 pathway [60].

Neuronal damage in ischemic injury is related to activation of apoptotic cascade after oxidative stress and/or mitochondrial. Caspase-3 is one of the important executors of apoptosis. A large body of evidence has showed that caspase-3 significantly increased after cerebral ischemia [61]. In a gerbil global cerebral ischemia/reperfusion injury model, Cao et al. found that baicalin remarkably inhibited the expression of caspase-3 at mRNA and protein levels by real-time RT-PCR and Western blot, respectively. Caspase-3 activity assay also elucidated that the administration of baicalin could significantly suppress caspase-3 in ischemic gerbil hippocampus [46].

Myeloid cell leukemia-1 (MCL-1) is a pro-survival member of the Bcl-2 family that is initially identified as an immediate-early gene expressed during differentiation of ML-1 myeloid leukemia cells and is considered as an anti-apoptotic gene. As a powerful co-activator of serum response factor (SRF), myocardia-related transcription factor-A (MRTF-A) is a major regulator of stimulus-dependent transcription of immediate-early genes (IEGs). MRTF-A has been found to play a key role in cell proliferation, differentiation, migration and apoptosis. A study has shown that SRF-MRTF-A/SRE-driven transcription was involved in promoting neuronal survival and inhibiting apoptosis induced by hypoxia/ischemia [62]. One study found that baicalin significantly increased the mRNA or protein levels of Bcl-2 and MCL-1in ischemic hemispheres of MCAO/R rats and in primary cortical neurons. Endogenous MRTF-A level was significant reduced in ischemia hemisphere of cerebral I/R rats, but markedly increased by baicalin in a dose-dependent manner. However, the anti-apoptosis effect of baicalin was significantly inhibited by transfection with the small interfering RNA of MRTF-A (MRTF-A siRNA) in primary cortical neuron cultures. The luciferase assays also indicated baicalin enhanced the transactivity of MCL-1 and Bcl-2 promoter by activating the key CArG box element, which was reduced by MRTF-A siRNA, suggesting MRTF-A may participate in the anti-apoptosis effect of baicalin, and MRTF-A was involved in the transcriptional activity of MCL-1 and Bcl-2 that was induced by baicalin [63].

Alteration of mitogen-activated protein kinases (MAPKs) signaling pathway is a key event in the apoptosis of cells. Mammals express at least three well-characterized subgroups of MAPKs: extracellular signal-regulated kinase (ERK), c-Jun N-terminal kinase/stress-activated protein kinase (JNK/SAPK) and p38. A study showed that excitotoxic-induced cell death of hippocampal neurons in mice lacking c-Jun N-terminal kinase 3 (JNK3) gene significantly decreased [64]. In addition, inhibition of extracellular signal-regulated kinases (ERK) and concurrent activation of JNK as well as p38 signaling pathways could induce apoptosis in rat PC12 cells [65]. Administration of baicalin by the dose of 200 mg/kg significantly increased the activation of ERK and diminished the activation of JNK and p38 in gerbils after ischemia/reperfusion, the total content of ERK, JNK and p38 remained unchanged [66].

## Inflammation

The inflammatory response during global cerebral ischemia/reperfusion also is one of the most important causes of neural damage [67]. Cyclooxygenase (COX) belongs to the family of prostaglandin peroxide synthases, and is a key enzyme in the catalytic conversion of arachidonic acid to prostaglandins and thromboxane. COX-2 is a key marker of inflammatory response, which can cause the neuronal death during the ischemia/reperfusion damage. Cheng et al. found that baicalin could reduce expression of COX-2 in the hippocampus of global ischemia rats. This finding indicates that the possible mechanism for the neuroprotective effects of baicalin may be related with inhibition of COX-2 expression following global ischemia [47].

Toll-like receptors (TLRs), which mediate the inflammatory reaction, are involved in the pathophysiological processes of ischemic brain injury [68]. TLR2 and TLR4 had been identified as key mediators of immune responses and inflammatory reactions related to Alzheimer’s disease, Parkinson’s disease, brain injury, and ischemic stroke, and they mediated proinflammatory responses through activating the nuclear factor kappa B (NF-κB) transcription factor. NF-κB is a key regulator involved in the inducible expression of proinflammatory mediators, such as inducible nitric oxide synthase (iNOS), cyclooxygenase-2 (COX-2), tumor necrosis factor-α (TNF-α), and interleukin-1β (IL-1β) [69]. Those factors and their reaction product nitric oxide (NO) and prostaglandin E2 (PGE2) are believed to be the major factors contributing to ischemic brain injury in post-ischemic inflammation. Therefore, TLR2/4 signaling pathway in the central nervous system is considered as the direct source of the damaging signal and an important potential therapeutic target [70]. Li et al. found that baicalin and its three analogs could inhibit the expression of TLR2 and TNFα in oxygen-glucose deprivation PC12 cell model. The results suggest the possibility of baicalin used in stroke therapy by targeting TLR2[40].

In another *in vivo* study, the effects of baicalin on TLR2/4 signaling pathway in a rat model of permanent focal cerebral ischemia were investigated. Adult Sprague-Dawley rats underwent permanent middle cerebral artery occlusion (MCAO). Baicalin was administered by intraperitoneally injected twice at 2 and 12 h after the onset of ischemia. Expression of TLR2/4, NF-κB, inducible nitric oxide synthase (iNOS), and cyclooxygenase-2 (COX-2) were determined by RT-PCR or western blot. NO and PGE2 production in rat brain were measured 24 h after MCAO. Serum content of tumor necrosis factor-alpha (TNF-α) and interleukin-1β (IL-1β) were detected by ELISA. The results showed that baicalin reduced the expression of TLR2/4 and NF-κB, decreased the expression and activity of iNOS and COX-2 in rat brain. Baicalin also attenuated the serum content of TNF-α and IL-1β. This study suggested that baicalin inhibits the TLR2/4 signaling pathway in cerebral ischemia [44]. As discussed above, the permanent NF-κB p65 activation is considered to contribute to infarction and cell death induced by ischemia reperfusion injury in both permanent MCAO animal models and stroke patients. Therefore, inhibition of permanent activation of NF-jB p65 can protect cerebral tissue from ischemic injury. Male Wistar rats were subjected to middle cerebral artery occlusion (MCAO) for 2 h followed by reperfusion for 24 h. Baicalin at doses of 50, 100 and 200 mg/kg was intravenously injected after ischemia onset. Twenty-four hours after reperfusion, the levels of NF-κB p65 in ischemic cortexes by Western blot analysis and RT-PCR assay were examined. The results showed that the nuclear NF-κB p65 expression was increased following ischemia reperfusion injury, indicating that a permanent NF-κB p65 activation occurred. Baicalin treatment effectively inhibited the permanent NF-κB p65 level by 73% and decreased the infarction area by 25% [44].

Cytosolic phospholipases A2 (CPLA2) is one of the super families of esterases that specifically hydrolyze the acyl ester bonding at the sn-2 position of membrane phospholipids including arachidonic acid. Increased cPLA2 activity generated proinflammatory lipid mediators, such as leukotrienes, eicosanoids, prostaglandins, and platelet-activating factor, and these factors played an important role in acute inflammatory responses and oxidative stress associated with neurological diseases [71]. P38MAPK is linked to the activation and phosphorylation of cPLA2 and arachidonic acid release. Baicalein could decrease the positive staining cells of phosphop38 MAPK and cPLA2 in the brain tissue after ischemia [43].

Intracellular Nod-like receptors (NLRs) which have rapidly emerged as central regulators of immunity and inflammation with demonstrated relevance to human diseases. Two members of this family, NOD1 (Nod-like receptor 1) and NOD2 (Nod-like receptor 2) can cause inflammation via activation of the NF-κB and MAP kinase pathways. Mammalian NOD2 seems to function as a cytosolic sensor for the induction of apoptosis [72]. Protein and mRNA of NOD2 were both highly expressed during the oxygen-glucose deprivation and reperfusion in BV2 cells, as well as in PC12 and primary neuron cells, which means the intracellular Nod-like receptors (NLRs) could be activated not only in microglia but in neuron. Baicalin could down regulate the expression of NOD2 and TNFα of the cells. In primary neuron cells and PC12 cells, baicalin’s effect on the protein expression of NOD2 could last for 6 h, but in BV2 cells its effect on the protein expression of NOD2 lasted only for 3 h, which suggested baicalin’s effect on BV2 cells in a temporary manner with difference from the primary neuron and PC12 cells. The effect of NOD2 and TNFα expression was highly correlated with the baicalin concentration in oxygen-glucose deprivation cells. The concentration of baicalin in the cells was increased 15 min after its administration. Meanwhile, the expression of NOD2 and TNFα was decreased 30 min after baicalin administration. This study showed baicalin played the role in protection of neuron damage, which was tightly related to expressions of the pattern recognition receptors, especially NOD2, in immune system [73].

Peroxisome proliferator-activated receptor γ (PPARγ) is a ligand-activated transcription factor that regulates lipid metabolism and glucose homoeostasis. Activation by its ligands, PPARγ translocate from cytoplasm into nucleus to regulate gene transcription. Nitration of PPARγ causes dysfunction of the transcription factor by inhibiting its agonist-stimulated nuclear translocation. PPARγ is also involved in the control of inflammation, in particular in modulating the production of inflammatory mediators. Using neuronal PPARγ deficient mice and PPARγ agonists, showed that PPARγ in neurons plays an intrinsic protective role in the brain against ischemia/reperfusion (I/R) injury. Activation of PPARγ by its agonist significantly reduced infarction size and improved neurologic function in a variety of animal models of stroke [74]. Following middle cerebral artery occlusion (60 min) and 2-24 hr reperfusion in rats, cerebral ischemia/reperfusion (I/R) induced up-regulation of PPARγ protein expression and translocation from the cytoplasm into the nucleus in a time-dependent manner were observed in a study. This study also demonstrated that the I/R-induced PPARγ alteration was reversed by baicalein. Baicalein treatment significantly inhibited the up-regulation of PPARγ expression and, furthermore, suppressed PPARγ nuclear accumulation as well as maintained PPARγ cytoplasmic retention [75].

## Excitotoxicity

Excitotoxicity is a key contributing factor during ischemia/reperfusion injury, in which disruption of GABAergic synaptic transmission plays an important role. Decrease of GABA_A_R-mediated inhibitory responses contributes to the ischemic damage. GABA_A_Rα1 and γ2 are two widely expressed subunits of GABA_A_Rs which gate a chloride channel to generate neuronal inhibition and then protect neurons from excitotoxic injury [76]. GABA_A_R-mediated chloride flux is depended on the intracellular Cl^-^ concentration which is regulated by the activity of K^+^-Cl^-^ (KCC) and Na^+^-K^+^-Cl^-^ (NKCC) families of cation chloride co-transporters. Decreased KCC2 and/or increased NKCC1 activity can lower GABAAR-mediated synaptic inhibition via elevating intracellular Cl^-^ concentration, and then making the neuron more vulnerable to ischemia [77]. Dai et al. found baicalin significantly increased the expressions of GABA_A_Rα1 and γ2 subunits at mRNA and protein levels in hippocampal CA1 subfield on an ischemia/reperfusion gerbil model. At the same time, protein level of KCC2 increased and NKCC1 decreased in baicalin-treated ischemic gerbils. These findings suggested that the neuroprotective effects of baicalin on gerbil global ischemia-induced neuronal injury are related to GABA_A_R-mediated inhibitory responses [66].

During and after ischemia, aspartic acid (Asp) and glutamate (Glu), the excitatory neurotransmitters (EAA), were released from neurons and astrocytes, which might contribute to the progressive neural injury, especially for glutamate [78]. It also has been reported that Glycine (Gly), Taurine (Tau) and γ-aminobutyric acid (GABA) are inhibitory neurotransmitters (IAA), which can inhibit the excess-activity of nerve cells caused by EAA [79]. A study demonstrated that baicalin administrated before ischemia restrained the increase of extracellular Asp and Glu during brain ischemia and early reperfusion and also increased the concentration of Gly, Tau and GABA in cerebrospinal fluid in MCAO rat [33].

Ischemic insults on neurons trigger excessive glutamate release that causes augmentation of Ca^2+^ resulting in excitotoxicity, Ca^2+^/calmodulin (CaM)-dependent protein kinase II (CaMKII) plays a key role in mediating some of the biochemical events leading to cell death following an acute excitotoxic insult [80]. CaMKII inhibition had protective effects on the neurons against an excitotoxic insult. The effects of baicalin on CaMKII were studied in global cerebral ischemia/reperfusion in gerbil and in cultured hippocampal and SH-SY5Y. The results showed that baicalin reversed the *in vitro* ischemia-induced increase of Ca^2+^ in cells and prevented increased phosphorylation levels of CaMKII induced by ischemia either *in vitro* or *in vivo* [41].

N-methyl-daspartic acid receptor (NMDARs), the most important excitatory amino acid (EAA) receptor, is a heteromeric composite composed by three kinds of subunits designated as NR1, NR2, and NR3, respectively. The up-regulation of NMDARs in neurons contributed to the increase of caspase-3 activity by inducing Ca^2+^ influx heavily [81]. Zhou et al. measured the expression of NMDAR1 in SH-SY5Y cells after glucose deprivation/reperfusion (OGD/RO) induced injury. The results showed OGD/RO contributed to the increase of NMDAR1 expression, while pretreatment with baicalin could reverse this phenomenon. This finding assumed that the neuroprotective effects of baicalin may be associated with the indirect inhibition of NMDAR induced EAA toxicity [82].

## Mitochondrial dysfunction

Mitochondria are the main production site for ATP in animal cells. He et al. investigated the effect of subacute baicalein treatment (30 or 100 mg/kg for 27days) on mitochondrial dysfunction induced by chronic cerebral hypoperfusion (CCR). Baicalein increased the respiration control ratio (RCR), the consumption of ADP, and the production of ATP. However, baicalein did not alter the O2 consumption [42]. The effects of baicalein alone (0.5-5.0 µM) on mitochondria isolated from rat brain were investigated by Li et al. The authors found that baicalein induced a decrease in the amount of O_2_ consumed in state 4 (respiration occurring in the absence of ADP or inhibitory agents) without altering ATP production, consequently increasing RCR and the mitochondrial P/O ratio [83].

## Blood-brain barrier permeability

The blood-brain barrier (BBB) is critical to control the exchange of materials between the peripheral circulation and the central nervous system (CNS), and very important structure to keep a homeostasis environment for proper function of CNS tissue. Cerebral microvascular endothelial cell via connecting with tight junction (TJ) complex is the basal structure of BBB [84]. Occludin is the most important tight junction protein to seal the tight junctions and degradation of occludin could induce to enhance the BBB permeability [85]. Matrix metalloproteinases (MMPs) is a family of proteolytic enzymes, in which MMP-9 is related to degrade occludin in the microvascular wall, resulting in an increase of the microvascular permeability and BBB disruption after ischemic stroke [86]. Tu et al. explored the effect of baicalin on the neuronal damage, brain edema and BBB permeability, then further investigated its potential mechanisms. In this study, baicalin was administered by intraperitoneal injection twice at 2 and 12 h after MCAO in rats. BBB permeability was measured 24 h following MCAO. Expression of MMP-9 protein and mRNA were determined by western blot and RT-PCR, respectively. Expression of occludin was detected by western blot. The results showed that BBB permeability were significantly reduced by baicalin. Elevated expression of MMP-9 protein and mRNA were significantly down-regulated by baicalin administration and MCAO caused the decreased expression of occluding, which was significantly up-regulated by baicalin administration. This study suggested that baicalin could inhibit MMP-9 expression and MMP-9-mediated occludin degradation and thus protect from ischemia injury in MCAO model [87].

Claudins are a family of transmembrane proteins that can form homodimers between neighboring cells. Claudin-5 is the main form expressed in cerebral microvascular endothelial cells. The function of claudins in the TJ complex is to limit paracellularion movement selectively, and this leads to a high electrical resistance across the barrier [88]. Zonula occludens (ZO) protein is able to interact with the C-terminal end of transmembrane proteins (e.g., claudins and occludin) in order to connect transmembrane proteins with the cytoskeleton. ZO plays a vital role in maintaining the continuity and integrity of TJ [89]. The TJ proteins (e.g., claudin-5, occludin and ZO-1) are phosphoproteins that exhibit rapid changes in their phosphorylation state. Phosphorylated TJ proteins undergo redistribution, which is a critical event leading to changes in the adhesive contacts between brain endothelial cells. PKCs are a family of serine/threonine kinases that regulate a variety of cell functions, some PKC isoforms have been shown to be critical for the phosphorylation of TJ proteins [90]. Under hypoxic and post-hypoxic reoxygenation conditions, PKC regulates TJ disassembly [91]. Zhu et al. found that after 24 h of OGD, transcription and expression of caludin-5 and ZO-1 in brain microvascular endothelial cells (BMVECs) was suppressed and the expression of PKC was significantly enhanced. This increase in PKC expression may also contribute to the rearrangement of TJ proteins such as claudin-5 and ZO-1 after phosphorylation, thereby resulting in damage to the BBB’s barrier function. However, baicalin treatment was effective in promoting the transcription and expression of claudin-5 and ZO-1 and decreased the level of PKC in endothelial cells and reduced the phosphorylation of TJ proteins under hypoxic stress. These results suggested that baicalin is capable of restoring the barrier function of the BBB under ischemic conditions and this beneficial effect may be linked to the decreased expression and phosphorylation of TJ proteins [92].

## Cellular toxicity of thrombin

Cellular toxicity of thrombin has been reported to be strongly associated with the nerve injury secondary to cerebral ischemia as thrombin could interact with the protease-activated receptor-1 (PAR-1) in brain tissues. Protease-activated receptors (PARs) belong to a superfamily of seven-transmembrane domain G-protein coupled receptors. Four subtypes of PAR members (PAR-1, 2, 3 and 4) have been identified, among which PAR-1 has been reported to be commonly distributed in the cerebral cortex, striatum, hypothalamus, hippocampus and cerebellum [93]. A study performed in adult male Sprague-Dawley rats indicated that thrombin is involved in the cerebral ischemic reperfusion injury through activating PAR-1. Some studies performed in null mice indicated that PAR1 increased infarct volume and caused neuronal damage after transient focal cerebral ischemia and combined cerebral hypoxia/ischemia [94]. Zhou et al. found that baicalin not only could significantly decrease the neurological score, but also down-regulated the levels of PAR-1 mRNA, PAR-1 and Caspase-3 after cerebral ischemic reperfusion injury in a MCAO rats model. These results demonstrated that baicalin might attenuate focal cerebral ischemic reperfusion injury through inhibition of PAR-1 and its apoptosis [95].

## Regulations of HSP70 expression

The heat shock proteins (HSPs) are ubiquitous and highly conserved molecular chaperones which are necessary for the proper folding of proteins. HSP70 is the major member of HSP family and has protective effects for the cells, because of protecting proteins from malfolding. During cerebral ischemia injury, overexpression of HSP70 could dramatically protect the neuron [96]. Dai et al. found that global ischemia resulted in a dramatic decrease of HSP70 protein expression in gerbil hippocampus, while baicalin treatment significantly increased HSP70 in the brain of gerbils with ischemia. These results suggest that enhancement in the expression of HSP70 protein may be involved in baicalin’s neuroprotection against ischemia/reperfusion injury [66].

## Regulations of brain-derived neurotrophic factor expression

BDNF is one of the most important neurotrophic factors which promotes neuronal survival and prevent cell damage following transient forebrain ischemia [97]. In a gerbil global cerebral ischemia/reperfusion injury model, Cao et al. examined the mRNA and protein expressions of BDNF in ischemic hippocampus by real-time RT-PCR and Western blot, respectively and found that treatment with baicalin remarkably promoted the expression of BDNF at mRNA and protein levels [46].

## Adult neurogenesis

Endogenous neural stem/progenitor cells (NPCs) may be therapeutic targets for promoting adult neurogenesis, brain plasticity and repair for the treatments of stroke [98]. Post-ischemic brains have the potential to stimulate proliferation and differentiation of NPCs and to induce direct migration of neuronal precursors toward ischemic areas, leading to replacement of damaged neurons [99]. The decision of cell fate into neurons or glia cells is one of the critical steps. Both Janus kinase/ signal transducer and activator of transcription 3 (Jak/stat3) and basic helix-loop-helix (bHLH) gene families appear to play a critical role in the decisions of cell fates during the differentiation of neural stem cells. Jak/stat3 could maintain the propagation and pluripotency of embryonic stem cells [100]. The bHLH gene family includes the repressor-type and the activator-type genes. Hairy enhancer of split 1 (Hes1) is a repressor-type bHLH gene, which is expressed in differentiating astrocytes in brain and the expression of Hes1 plays a critical role in the formation of astrocytes [101]. Mash1 and NeuroD1 are activator-type bHLH genes, which are expressed in differentiating neurons [102]. The effects of baicalin on neuronal differentiation of NPCs were studied on embryonic NPCs from the cortex of E15-16 rats. The NPCs were treated with baicalin (2, 20 μM) for 2 h and 7 days. Neuronal and glial differentiations were identified with mature neuronal marker microtubule associated protein (MAP-2) and glial marker Glial fibrillary acidic protein (GFAP) immunostaining fluorescent microscopy respectively. Phosphorylation of stat3 (p-stat3) and expressions of bHLH family genes including Mash1, Hes1 and NeuroD1 were detected with immune-fluorescent microscopy and Western blot analysis. The results revealed that baicalin treatment increased the percentages of MAP-2 positive staining cells and decreased GFAP staining cells. Meanwhile, baicalin treatment down-regulated the expression of p-stat3 and Hes1, but up-regulated the expressions of NeuroD1 and Mash1. Those results indicated that baicalin can promote the neural differentiation but inhibit glial formation and its neurogenesis-promoting effects are associated with the modulations of stat3 and bHLH genes in neural stem/progenitor cells [103].

## Pharmacological signaling pathway networks analysis

Gene expression profiling is the measurement of the activity (the expression) of thousands of genes at once, to create a global picture of cellular function. A high-density cDNA microarray was used to explore the differential gene expression and the pharmacological mechanism of baicalin in focal cerebral ischemia in MCAO rats. Gene expression was demonstrated using a “Biostar40S” gene microarray. Semi-quantitative reverse transcription-polymerase chain reaction (RT-PCR) was used to verify the result of the selected genes. Results showed that baicalin reduced the infarction areas in focal cerebral ischemia rats and 89 genes showed increased expression and 88 genes simultaneously showed decreased expression. These genes are involved in metabolism, signal transduction, cell organization, responses to stress, and transcription regulators.

### Metabol

Metabolism-related genes showed prominent changes in expression. These included ADP-ribosylation factor, enolase, adenine phosphoribosyl transferase, and palmitoyl-protein thioesterase. In addition, histamine Nmethyl transferase simultaneously showed decreased expression. The largest increases in expression were shown by genes involved in protein tyrosine phosphatase receptor type D and receptor type A. S100 calcium binding protein A9 also showed prominent changes. In addition, the expression of protein kinase C-binding protein Zeta and surfactant-associated protein decreased. Protein kinase is controlled by specific binding proteins, which are believed to sequester each type of kinase to the region of a neuron, such as the postsynaptic specialization or cell nucleus, required for its function [104]. The protein kinase C binding protein Zeta is one of these proteins. Arachidonic acid epoxygenase, an adapter protein of the prostaglandin and leukotriene family of intracellular messengers, also appears to play an important role in the regulation of signal transduction in the brain and elsewhere [105]. The expression of nucleolar phosphoprotein p130, an adapter protein that participates in nucleolar disassembly and cell cycle, decreased. In addition, peroxisomal membrane protein showed increased expression over this interval. Two genes related to the transcription regulator were altered. These were spliceosome associated protein and DNA primase. Differential gene expression showed that baicalin played an important role in cell signal transduction and protein phosphorylation after MCAO, and might act as a neuronal protectant [11].

Proteomics is an efficient method for the investigation of expression levels of multiple proteins synchronously. Zhang et al. applied proteomics to investigate the different protein expression modes in mice brains after middle cerebral artery occlusion (MCAO) with or without administration of baicalin. Twenty-four proteins which had a 3-fold change in abundance compared to the sham control sample were selected to be identified. Gene Ontology analysis linked these proteins to fifteen biological processes, including cellular process, developmental process and biological regulation. When comparing the different protein expression modes between baicalin treatment MCAO group and non-drug treatment MCAO group, the results showed that baicalin was effective in regulating the protein expressions. Proteins in the energy metabolism system were regulated better than neurogenesis and apoptosis [106].

Several contemporary analytical tools have been developed to determine trends and to assimilate and visualize molecular interaction data. These tools, coupled with computerized databases, allow a better understanding of the gene interactions that cumulatively affect important biological pathways. In order to understand the signaling network pathways associated with baicalin-related biological effects. MCAO-induced mice received baicalin 5mg/Kg, controls received vehicle only. Following ischemia-reperfusion, Array Track analyzed the whole genome microarray of hippocampal genes (16,463 genes), and MetaCore analyzed differentially expressed genes. Enrichment analysis identified 10 significant biological processes in baicalin and controls. Of the 10 most significant molecular functions, 7 were common to baicalin and controls, and 3 occurred in baicalin. Results showed that baicalin pathways most apparently influenced by gene expression were associated with cytoskeleton remodeling, cellular development, Vascular endothelial growth factor (VEGF) signaling via VEGF receptor 2, and Tumor growth factor (TGF)-beta-dependent epithelial-mesenchymal transition (EMT) induction via MAPK. Baicalin treatment induces gene expression that not only prevents apoptosis but also promotes oligodendrocyte survival and myelination signaling. It was also found that baicalin acted on calcium ion (Ca^2+^)-dependent signaling cascades, shown to play a neuroprotective role in cerebral ischemia by signallingCa2+/CaM dependent ERK activation. Treatment with baicalin was shown to increase the network processes for the anti-apoptotic p21 and mothers against decapentaplegic homolog 3 (SMAD3). Baicalin activation of the cyclinD-SMAD3-TGF beta network pathway may improve wound healing after cerebral ischemia. In general, this study showed that the clinical effectiveness of baicalin was based on the complementary effects of multiple pathways and networks [107].

## The difference between baicalin and baicalein on neuroprotective effects

Baicalein is an aglycon derivative from baicalin. Not only there are structural similarities between these two flavonoids, but also, they can convert to each other during the metabolism in the body. However, baicalin and baicalein still exert different effects on neuronal cells. Gao et al. studied the effects of four major flavonoids present in *Scutellaria baicalensis Georgi* on hydrogen peroxide-induced neuronal cell damage on human neuroblastoma SHSY5Y cells and found that baicalein offered the strongest protection against H_2_O_2_-mediated cytotoxicity. On the other hand, the flavonoids at high concentrations exhibited high toxicity to SH-SY5Y cells, the concentrations for 50% cell death of baicalein and baicalin were 320 and 390 µM, respectively [108]. In another study, the neuroprotective effects of baicalin and baicalein against glutamate/NMDA (Glu/NMDA) stimulation and glucose deprivation were investigated in primary cultured rat brain neurons. It was found that both baicalin and baicalein significantly reduced Glu/NMDA-increased LDH release, in which baicalein is much more potent than baicalin and only baicalein did moderately decrease Glu/NMDA-induced nitric oxide production. In the glucose deprivation (GD) study, baicalein but not baicalin showed significant protective effects on the GD-increased LDH release in cultured rat brain neurons. These results suggest that baicalein is better than baicalin in preventing neurotoxicity induced by both glutamate and GD [109]. Li et al also confirmed that baicalein exhibited higher toxicity to normal PC12 cells, and it also showed stronger protective effect in PC12 cell with oxygen-glucose deprivation than baicalin [40].

**Figure 2.**
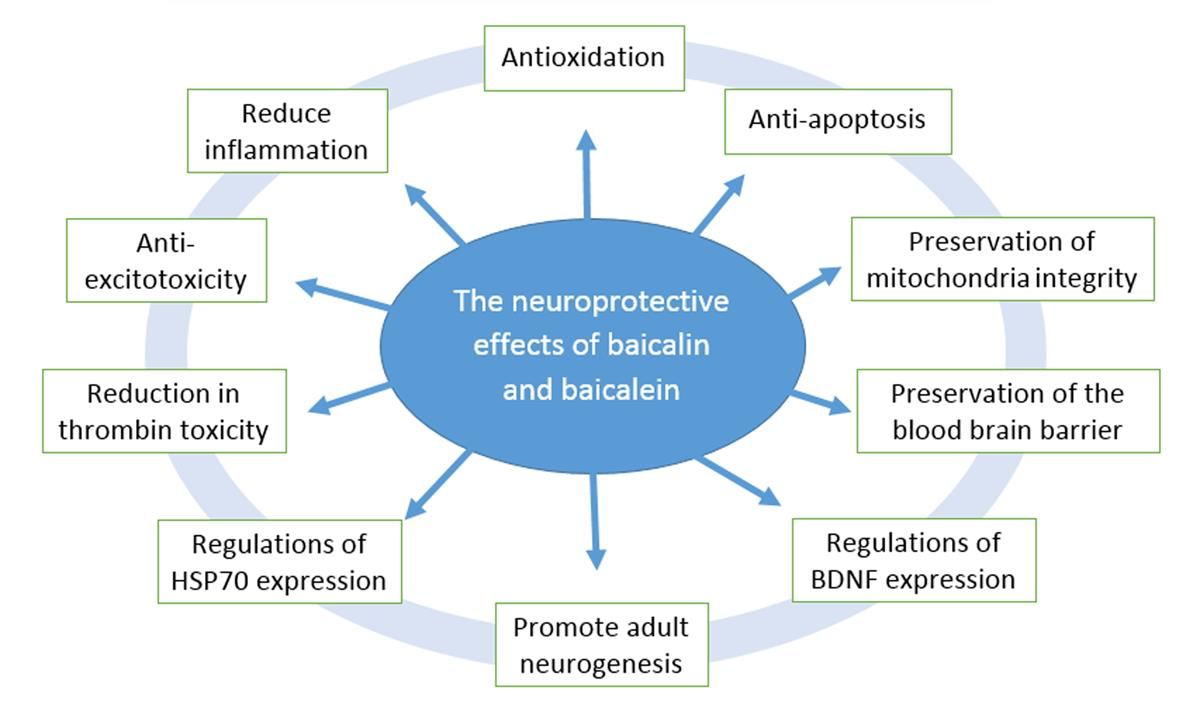
The neuroprotective effects of baicalein and baicalin.

## Conclusion

Traditional Chinese medicine is one of the world's oldest documented medical systems based on herbal remedies. *Scutellaria baicalensis*, the most widely used herb, has been in use for thousands of years. Baicalin and its aglycon baicalein are the principal components found among other flavonoid derivatives in the roots of *Scutellaria baicalensis Georgi*. Abundant scientific evidence shows that the neuronal protective effects of baicalin and baicalein are related to anti-oxidant, anti-apoptotic, anti-inflammatory and anti-excitotoxicity effects, protection of the mitochondria, promotion of neuronal protective factors expression and adult neurogenesis and other factors (Fig. 2). As the multi-target neuroprotective agents, further vigorous research, including enhancement of the bioavailability and clinical research on ischemic stroke, is needed. These substances can also be used as leading compounds to develop potent novel neuroprotective agents. According to the published data collected in this review, we can reasonably speculate that some of the effects are direct and some are indirect. To improve the efficacy of neuroprotection, we must clearly understand the targets of these effects. Recently, bioinformatics and computer-aided drug discovery/design methods have played a major role in the development of therapeutically important small molecules. We suggest that the exact pharmacological targets of baicalin and baicalein for neuroprotection would be clarified with the aid of bioinformatics methods, and then we could design and develop more effective neuroprotective agents accordingly.

No. 7162079 (PI: Wenqiang Chen), the Beijing Natural Science Foundation, No.7172094 (PI: Xiaobo Huang), and the Beijing Talents Project (PI: Wenqiang Chen).
